# Does change in area-level deprivation, change health outcomes? A latent class growth analysis of population data.

**DOI:** 10.23889/ijpds.v9i5.2688

**Published:** 2024-09-18

**Authors:** Finola Ferry, Ron McDowell, Michael Rosato, Jamie Murphy, Gerard Leavey

**Affiliations:** 1 Ulster University; 2 Administrative Data Research Centre Northern Ireland

## Background

While the link between deprivation and health is established, little is known about the association of persistent deprivation or deprivation trajectories over time with adverse health outcomes.

## Aim

To examine latent class trajectories of area level-deprivation from 2010-2016 and their association with social distress indicators from 2017-2021.

## Method

Analysis was based on linked administrative data (N=1,569,110) from NI GP Registrations Index, emergency department records (2017-21), enhanced prescribing data (2017-21) and biennial area-level deprivation (2010-2016). Latent class growth analysis (LCGA) identified deprivation trajectories between 2010 and 2016; and regression-based models, adjusted for age, sex and urbanicity, examined their association with indicators of individual level distress: occurrence of death, any accident and emergency (A&E) visit, and any psychotropic medication between 2017 and 2021. 

## Results

Based on comparison of models and fit statistics, a non-linear LGCA model identified six trajectories: consistently deprived (21.2%); slight improvement (7.1%); substantial improvement (1.2%); consistently average deprivation (31.7%); slight worsening (18.5%); and consistently non-deprived (20.4%). Consistently living in a deprived area was associated with higher likelihood of death, A&E visits and receipt of hypnotics, anxiolytics, and anti-depressants. Compared to consistent deprivation, slight improvement was associated with lower likelihood of death (OR=0.94, CI: 0.91-0.97) and all psychotropic medication in the follow-up period.

## Implications

Findings indicate that even a slight improvement in deprivation status can have a positive influence on health outcomes. Further population-wide analysis of deprivation trajectories, their determinants and associated health impact will inform targeted interventions that promote social mobility and improved health.

